# Porous Hierarchical Ni/Mg/Al Layered Double Hydroxide for Adsorption of Methyl Orange from Aqueous Solution

**DOI:** 10.3390/nano13131943

**Published:** 2023-06-26

**Authors:** Tayyaba Waheed, Salah ud Din, Lei Ming, Pervaiz Ahmad, Pu Min, Sirajul Haq, Mayeen Uddin Khandaker, Imed Boukhris, Mohammad Rashed Iqbal Faruque, Fazal Ur Rehman, Israf Ud Din

**Affiliations:** 1 State Key Laboratory of Chemical Resource Engineering, Beijing Engineering Center for Hierarchical Catalysts, Beijing University of Chemical Technology, No. 15 Beisanhuan East Road, Chaoyang District, Beijing 100029, China; 2 Department of Chemistry, University of Azad Jammu and Kashmir, Muzffarabad 13100, Pakistan; 3 Department of Physics, University of Azad Jammu and Kashmir, Muzaffarabad 13100, Pakistan; 4 Center for Applied Physics and Radiation Technologies, School of Engineering and Technology, Sunway University, Bandar Sunway 47500, Selangor, Malaysia; 5 Department of General Educational Development, Faculty of Science and Information Technology, Daffodil International University, Dhaka 1341, Bangladesh; 6 Department of Physics, Faculty of Science, King Khalid University, P.O. Box 9004, Abha 62217, Saudi Arabia; 7 Space Science Centre, Universiti Kebangsaan Malaysia (UKM), Bangi 43600, Selangor, Malaysia; 8 Department of Chemistry, College of Science and Humanities, Prince Sattam Bin Abdulaziz University, P.O. Box 173, Al-Kharj 16278, Saudi Arabia

**Keywords:** calcination, waste water, characterization, adsorption, isotherm, kinetics

## Abstract

A basic urea technique was successfully used to synthesize Mg/Al-Layered double hydroxides (Mg/Al LDHs), which were then calcined at 400 °C to form Mg/Al-Layered double oxides (Mg/Al LDOs). To reconstruct LDHs, Mg/Al LDOs were fabricated with different feeding ratios of Ni by the co-precipitation method. After synthesis, the Ni/Mg/Al-layered double hydroxides (NMA-LDHs) with 20% and 30% Ni (S1 and S2) were roasted at 400 °C and transformed into corresponding Ni/Mg/Al-layered double oxides (NMA-LDOs) (S1a and S2b, respectively). The physiochemical properties of synthesized samples were also evaluated by various characterization techniques, such as X-ray diffraction (XRD), Scanning electron microscopy (SEM), Energy dispersive X-ray spectroscopy (EDS), Fourier transform infrared (FTIR), and Brunauer, Emmett, and Teller (BET). The adsorption behavior of methyl orange (MO) onto the synthesized samples was evaluated in batch adsorption mode under varying conditions of contact time, adsorbent quantity, and solution pH. As the dosage amount increased from 0.01–0.04 g, the removal percentage of MO dye also increased from 83% to 90% for S1, 84% to 92% for S1a, 77% to 87% for S2, and 93% to 98% for S2b, respectively. For all of the samples, the adsorption kinetics were well described by the pseudo-second-order kinetic model. The equilibrium adsorption data were well fitted to both Langmuir and Freundlich models for methyl orange (MO). Finally, three adsorption-desorption cycles show that NMA-LDHs and NMA-LDOs have greater adsorption and reusability performance for MO dye, signifying that the design and fabrication strategy can facilitate the application of the natural hydrotalcite material in water remediation.

## 1. Introduction

Water is essential for the development and existence of human society. However, water pollution has become a serious problem with the increase in global development. The physiochemical properties of fresh water can be affected by industrial waste water discharges having color impurities, suspended solids, dissolved organics, and salts [1]. The production of dyes in the United States, Western Europe, and Japan has diminished considerably, while production in China, India, and some South Asian countries has increased over the past 25 years [2]. Pigments in water are considered a primary pollutant in contaminated water. When dyes are discarded into waste water, they become inert, non-biodegradable, mutagenic, and carcinogenic for humans. Hence, one of the main environmental concerns is to somehow remove these dye effluents. Therefore, it becomes important to explore methods that eliminate contaminants from waste water efficiently and without damaging the environment. Consequently, wastewater treatment of the dyes is a field of interest for many researchers around the globe in an effort to develop a sustainable treatment strategy [3]. Many different physiochemical techniques have been established for this purpose [4,5], i.e., adsorption [6,7], biological oxidation [8], photocatalysis [9,10], chemical coagulation [11], and ion-exchange [12]. The most popular technique for eliminating dyes from wastewater is adsorption. Its advantages over other methods are its versatility, simplicity of use, low volume of sludge, and easy setup. In recent years, much attention has been paid to the removal of dyes by using different adsorbents [13]. 

Methyl orange (MO) is one of the anionic dyes that is mostly used for coloring fabrics in the textile industry. MO can cause genuine issues in people as well as in animals, i.e., skin allergies, and if ingested, it can cause gastrointestinal irritation with nausea, vomiting, diarrhea, sickness, and respiratory irritation. Many researchers have worked over the last few decades to investigate various absorbents to remove such toxic dyes, such as activated carbon [14], graphene [15], fly ash [16], microorganism-graphene oxide [17], and biomasses [18]. Unfortunately, all of these treatments have drawbacks due to the difficulty of the regeneration process and their high cost [1]. Therefore, it is very important to introduce a system that degrades and decolonizes pollutants like dyes from wastewater before discharge. 

One such popular class of material is Layered double hydroxide. The general formula for layered double hydroxides (LDHs), commonly referred to as hydrotalcite-like anionic clay, is [M^2+^_(1−x)_ M^3+^_x_ (OH)_2_]^x+^ (A^n−^)_(x/n)_ × mH_2_O. Since it has a layered lamellar structure, is porous, has a high surface area, and has exchangeable interlayer anions, mH_2_O has been reported to be an effective adsorbent material for treating wastewater [19]. LDHs are preferred over other adsorbents due to their low cost, porous structure, stability, and large adsorption capacity for the removal of pollutants, including wastewater treatment. Hence, the use of LDHs in wastewater treatment is very beneficial. Different LDHs have been reported, such as Mg-Al LDH [20], Fe_3_O_4_@MgAl-LDH [21], and Mg-Al-CO^3^−LDH [22], and have been utilized for the removal of dyes from waste water. For example, Lu et al. prepared the microsphere Fe_3_O_4_@MgAl LDH via the solvothermal route and observed its adsorptive removal properties for Congo red dye [21]. Purushothaman et al. synthesized Ni-Al LDH by the co-precipitation method, and adsorption results showed that the calcined Ni-Al LDH can be employed as an adsorbent for the removal of dye from aqueous solutions [23]. Mg-Fe-CO^3^−LDH was synthesized by Ahmed et al., who also looked into the adsorption of anionic reactive dye [24]. Tri-metallic LDHs have been studied to increase the amount of anions and enlarge host layers. Kowalik et al. synthesized CuZnAl-LDH and investigated its memory effect by XRD studies [25]. Chagas et al. used the hydrothermal method to prepare MgCoAl and NiCoAl LDHs and studied the structural characterization and thermal decomposition of these samples [26]. Ni et al. synthesized Zn/Al-LDH and used its calcined product for the adsorption of MO dye from its aqueous solution [1]. Ai et al. used the hydrothermal method to synthesize Mg/Al-LDH to examine the adsorption of MO dye [27]. Monash et al. prepared Ni/Al-LDH by a simple co-precipitation method from their nitrate salts and used it as an adsorbent to eliminate methyl orange (MO) dye from wastewater [23]. 

In this article, we reported a new approach to fabricating Mg/Al LDH with different Ni concentrations (20% and 30%) (S1 and S2) and their calcined products (S1a and S2b), as by inserting Ni into LDHs, MO dye can be removed from aqueous solutions with increased surface area, chemical affinity, structural stability, and electrostatic interaction than with simple LDHs. To optimize the sorption process, the effects of several parameters, such as solution pH, dye concentration, amount of dosage, and contact duration, were investigated. The kinetics and isotherm processes were studied through batch adsorption experiments. Furthermore, the as-prepared samples were characterized by different techniques, i.e., FTIR, XRD, SEM, EDS, and BET, to carefully examine the structural relationship between porosity and the adsorption amount of MO in aqueous solution.

## 2. Experimental

### 2.1. Materials

Magnesium chloride (MgCl_2_·6H_2_O), aluminum chloride (AlCl_3_·6H_2_O), urea [CO(NH_2_)_2_], nickel nitrate (Ni(NO_3_)_2_·6H_2_O), sodium hydroxide (NaOH), and MO were purchased from Xilong Science Co., Ltd. (Tianjin, China). All of the chemical reagents used in the research were analytical grade and did not require any additional purification. Deionized water was used in every step of the experiment.

### 2.2. Synthesis

Triple-metallic Ni/Mg/Al (20% and 30% Ni) LDHs were synthesized by the hydrothermal method followed by the co-precipitation method, which is different from the reported work in which NMA-LDH was synthesized via the single-step hydrothermal method [28]. In detail, first, we prepared carbonated hydrotalcite Mg/Al with a molar ratio of 2:1 by mixing MgCl_2_·6H_2_O, AlCl_3_·6H_2_O, and NaOH in 100 mL of deionized water. After mixing in a ball milling apparatus, the obtained mixture was poured into a 100-milliliter Teflon-lined stainless steel autoclave containing 0.18 moles of urea. The autoclave was maintained at 150 °C for 12 h, and then the resulting precipitates were left to cool at room temperature. The resulting precipitates were washed five times with water and ethanol. Finally, it was dried for 12 h at 80 °C and then roasted at 400 °C to prepare LDOs [29]. In the process of recovery, 20% Ni and 30% Ni were added to prepare tri-metallic LDHs by the co-precipitation method. The following is a description of the potential reaction mechanism [29,30]:

(1) $$CO{\left(NH_{2}\right)}_{2}+3H_{2}O\to 2OH^{-}+2NH_{4}^{+}+CO_{2}$$

(2) $$CO_{2}+2H_{2}O\to CO_{3}{}^{2-}+2H^{+}$$

(3) $$Mg^{2+}+Al^{3+}+OH^{-}+CO_{3}^{2-}\to MgAl-CO_{3}^{2-}-LDH$$

(4) $$MgAl-CO_{3}^{2-}-LDH\overset{400\;{}^{\circ}C}{\to }MgAl-LDO+H_{2}O$$

(5) $$Ni^{2+}+MgAl-LDO+OH^{-}\to NiMgAl-LDH$$

(6) $$NiMgAl-LDH\overset{400\;{}^{\circ}C}{\to }NiMgAl-LDO+H_{2}O$$

Under hydrothermal conditions, urea can be broken down into ammonia gas (NH_4_^+^) and carbon dioxide (CO_2_), which can subsequently be changed into CO_3_^2−^ and OH^−^ by the addition of water. Finally, NiMgAl-LDH was formed after the interaction between OH^−^ and metal cations.

**Figure 1:**
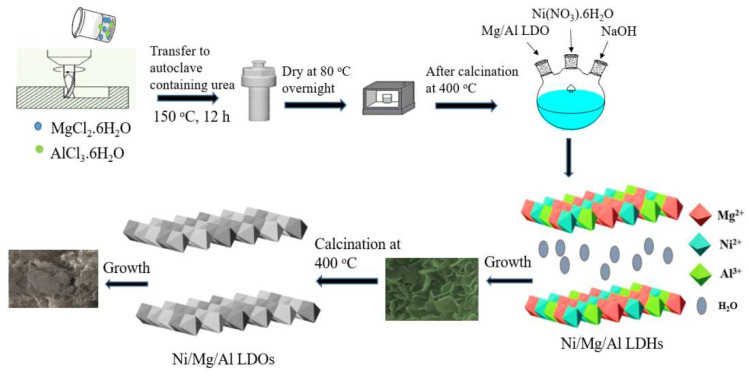
NMA-LDHs and NMA-LDOs synthesis scheme.

A schematic illustration of the synthesis of NMA-LDHs and NMA-LDOs is presented in Figure 1. The percentages of Ni (20% and 30%) added were determined according to the proportion of Mg content in LDOs, and the final products were labeled as NMA-LDHs with 20% Ni as S1, NMA-LDHs with 30% Ni as S2, NMA-LDOs with 20% Ni as S1a, and NMA-LDOs with 30% Ni as S2b.

### 2.3. Characterizations

The X-ray diffraction (XRD) pattern of the samples was obtained on an X-ray diffractometer (Rigaku D8 ADVANCE X-ray, Tokyo, Japan) using a Cu Kα radiation source (λ = 1.5406 Å). Using a field emission scanning electron microscope (SEM-Hitachis-4800, Tokyo, Japan) fitted with an energy-dispersive X-ray spectroscopy (EDX) instrument (EDAX SDD Octane Super, Wiesbaden, Germany), elemental mapping analyses were carried out. To calculate the specific surface area, porosity, and pore diameter, nitrogen adsorption/desorption were performed on the volumetric analyzer(Micrometrics ASAP 2460, Norcross, GA, USA) at 77 K. Fourier transform infrared spectroscopy (FT-IR Nicolet 8700, Waltham, MA, USA) was used for functional group analysis. The MO concentration was quantitatively tested after microfiltration by a UV-Vis spectrophotometer (Shimadzu UV-2501 PC, Tokyo, Japan).

### 2.4. Batch Adsorption Experiment

Adsorption studies were carried out to investigate the effects of contact time, pH, and adsorbent dosage. These tests were performed using 10 mg of each prepared material in a 250-milliliter beaker that contained 100 mL of a 25 mg/L MO solution at a solution pH that ranged from 2 to 12. The pH of the solution was adjusted to suitable values by adding NaOH (0.1 M) or HNO_3_ (0.1 M) and measured using a GLP 21 pH meter. The suspension was collected every five minutes, and the final MO concentration was measured using a UV-visible spectrophotometer with a maximum 464 nm wavelength. The effect of dosage was examined by mixing MO solution (25 mg/L) and varying the adsorbent dosage (10–40 mg) of each prepared sample in a 250-milliliter beaker.

According to the following equation, the adsorption capacity (mg/g) and percent removal efficiency (%) were determined:

(7) $$\mathrm{Removal}\;\%=\frac{\left(C_{o}-C_{e}\right)}{C_{e}}\times 100$$

whereas *C_o_* is the initial and *C_e_* is the equilibrium concentration of MO solution.

All experiments were performed in triplicate. Experiments were conducted in series under the same conditions to observe the adsorbent’s reusability. The suspensions were carried out after equilibrium time, washed with 8% HNO_3_ to get rid of any remaining MO, and dried in an oven at 100 °C before performing another adsorption cycle.

### 2.5. Adsorption Kinetics

Adsorption kinetic tests for MO were investigated by adding 10 mg of each sample individually to 100 mL of MO solution (25 mg/L) at pH 9. The beaker was airtight and continuously stirred at room temperature. After every 5 min, the aqueous sample was taken out, and the concentration of MO dye was calculated by using a UV spectrophotometer (Shimadzu UV-2501 PC, Tokyo, Japan) at a maximum absorption wavelength of MO (λ_max_ = 464 nm). The adsorbed quantity (*q_t_*) of MO on prepared samples was calculated using Equation (8).

(8) $$q_{t}=\frac{(C_{o}-C_{t})\times V}{m}$$

While *C_o_* (mg/L) is the initial concentration and *C_t_* (mg/L) is the concentration of MO dye at contact time, *V* (L) is the volume of solution, and *m* (g) is the mass of the adsorbent, *q_t_* (mg/g) is the quantity adsorbed at contact time.

To understand the controlled mechanism of the adsorption process, the pseudo-first-order and pseudo-second-order kinetic models were applied [31,32]. These kinetic model linear expressions are given in Equations (9) and (10), respectively. In these equations, *q_e_* (mg/g) represents the amount adsorbed at equilibrium, *q_t_* (mg/g) represents the amount adsorbed at contact time *t*, *k*_1_ (min^−1^) represents the pseudo-first-order constant, and *k*_2_ (g/mg min) represents the pseudo-second-order kinetic rate constant.

(9) $$\ln(q_{e}-q_{t})=\ln q_{e}-k_{1}t$$

(10) $$\frac{t}{q_{t}}=\frac{1}{k_{2}{q_{e}}^{2}}+\frac{t}{q_{e}}$$

### 2.6. Adsorption Isotherm

For the calculation of the remaining MO concentration using a UV spectrophotometer. An 0.01 g aliquot of each sample was dispersed in 100 mL of MO solutions (5–25 mg/L) at pH 9 for 80 min at constant stirring, and at 25 °C, a sample of 2 mL of the resultant suspension was obtained in order to determine the remaining concentration of MO by using a UV spectrophotometer. Equation (8) was used to calculate the corresponding removal capacity of each sample for MO. Using Equations (11) and (12), the acquired data were fitted to the Langmuir and Freundlich isotherm models, where *C_e_* (mg/L) denotes the equilibrium concentration, *q_e_* (mg/g) is the amount adsorbed at equilibrium, and *q*_max_ is the possible maximum amount adsorbed (mg/g). Theoretically, *K_L_* denotes the Langmuir constant (L/mg), *K_F_* denotes the Freundlich constant [(mg/g) (L/mg)^1/*n*^], and the heterogeneity factor is denoted by 1/*n* [33]. The maximum removal capacity for the Freundlich model can be calculated from Equation (13).

(11) $$\frac{C_{e}}{q_{e}}=\frac{C_{e}}{q_{\max}}+\frac{1}{q_{\max}K_{L}}$$

(12) $$\ln q_{e}=\ln K_{F}+\frac{1}{n}\ln C_{e}$$

(13) $$K_{F}=\frac{q_{m}}{C_{o}{}^{1/n}}$$

The shape of the Langmuir isotherm and nature of adsorption can be studied by using the separation factor *R_L_*, which has the following expression:

(14) $$R_{L}=\frac{1}{1+K_{L}C_{o}}$$

where *C_o_* (mg/L) is the initial MO concentration and *K_L_* denotes the Langmuir isotherm adsorption constant (L/mg).

### 2.7. Regeneration Cycle

The reusability study of the adsorbents is an important aspect of their practical application. Adsorption-desorption treatment cycles were performed to assess the reusability of the prepared samples. 1 g of the samples was added to 50 mL of 25 mg/L MO solutions, and the reaction was shaken at 150 rpm. Samples were collected after the MO dye adsorption; they were cleaned to remove any remaining MO using 20 mL of 8% HNO_3_. To eliminate excess acid, the samples were washed many times with distilled water and then dried at 100 °C before being applied in the next adsorption cycle. The adsorption-desorption operations were carried out again with the same adsorbent [34].

## 3. Results and Discussion

### 3.1. Crystal Structure

**Figure 2:**
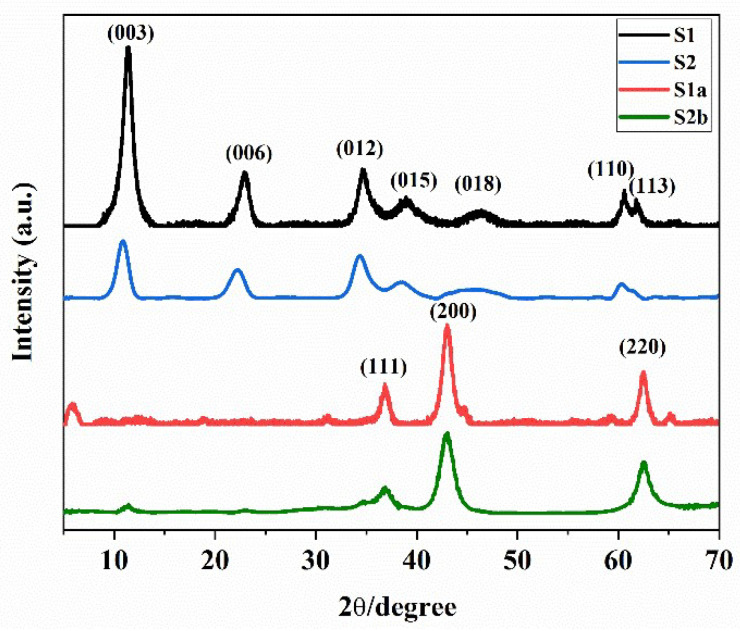
XRD patterns for S1, S2, S1a, and S2b samples.

The powder XRD pattern shows strong reflections of hydrotalcite for samples S1 and S2, with seven significant peaks placed at approximately 11.3°, 22.98°, 34.6°, 39.12°, 46.3°, 60.62°, and 62.3° with the series of 003, 006, 012, and 015 planes of Mg/Al hydrotalcite (JCPDS 35-0965) and planes of takovite (JCPDS 15-0087) with the series of 018, 110, and 113, respectively (Figure 2). The obtained values are also in good agreement with the literature [35]. No peak of another phase was found, indicating the purity of the synthesized samples. After calcination, the S1 and S2 change into their corresponding multi-metal oxides, or LDOs. S1a and S2b samples exhibit a series of reflections at 111, 200, and 220, located at approximately 36.8°, 43.0°, and 62.5° [36]. Moreover, the XRD patterns for S1a and S2b are in accordance with the corresponding Ni/Mg/Al LDOs [28].

### 3.2. Morphology and Elemental Composition

**Figure 3:**
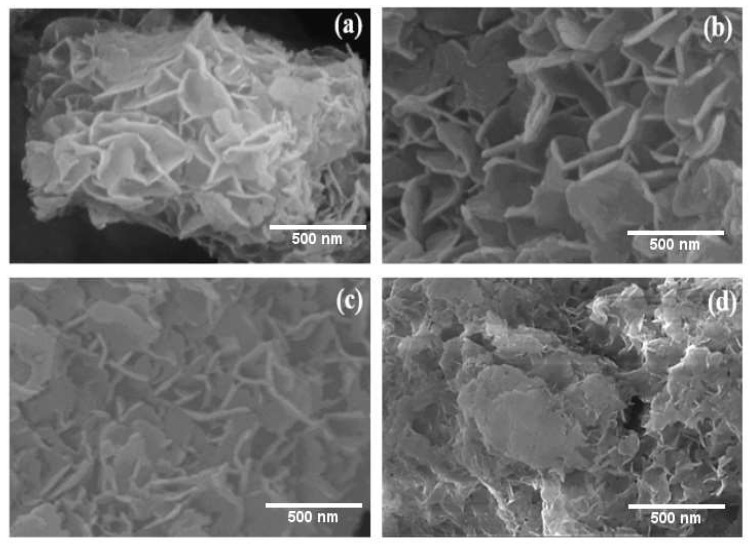
SEM images of S1 (**a**), S2 (**b**), S1a (**c**), and S2b (**d**).

Figure 3a–d displays the SEM images and associated mappings of the samples, highlighting the ultrathin, layered hexagonal structure [37]. The S1 and S2 are homogeneously grown in a lamellar structure (Figure 3a,b). No other morphology can be found from the SEM images, which suggest Ni^2+^ inserts into the hydrotalcite crystal successfully. One observes the hierarchical abundance of porous structures for S1a and S2b (Figure 3c,d), which is favorable for enhancing the adsorption. The lamellar sheet’s various pore densities produce a range of pore diameters, which likely helps to increase removal capacity and improve surface area utilization. The difference in pore density of the lamellar sheet results in different pore sizes, which probably enhances the removal capacity and particularly improves the utilization efficiency of the surface area. More active sites for the adsorption of MO dye can be provided by the lamellar mesoporous structure’s high surface area and hierarchical pore structure, and the interconnected pore structure system can increase the adsorption. Moreover, the presence of Ni in LDHs may be more effective in eliminating anions with lower ionic radii since these can more easily fit into the interlayer gap of LDHs.

**Figure 4:**
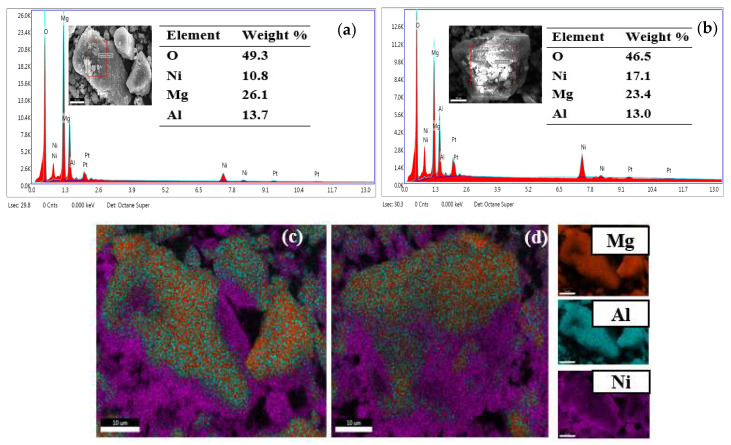
EDS spectra of S1a (**a**), EDS spectra of S2b (**b**), elemental mapping images of S1 (**c**), and S2 (**d**).

The EDS spectrum (Figure 4a,b) shows S1a and S2b to be composed of Ni, Mg, Al, and O, containing the equivalent weight percentages of 10.8, 26.1, 13.7, 49.3, and 17.1, 23.4, 13.0, and 46.5, respectively. In the S2b sample, the amount of Ni is higher as compared to the S1a sample. Therefore, the S2b sample is Ni-enriched in the core.

The elemental mapping of S1 and S2 in Figure 4c,d shows that Ni occupies the gaps between the initial Mg-Al structures.

### 3.3. Pore Properties

**Figure 5:**
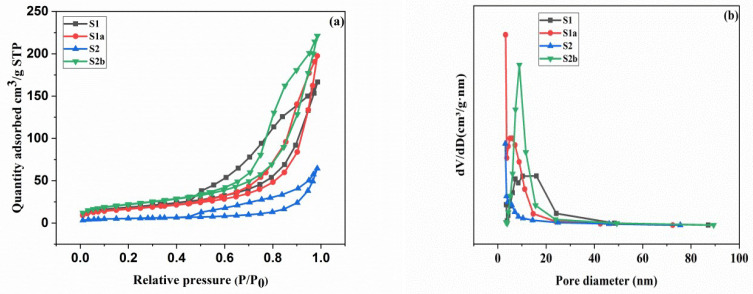
Nitrogen adsorption-desorption isotherms (**a**) and pore size distribution curves (**b**) of the prepared samples.

The corresponding N_2_ adsorption-desorption isotherms and pore size distribution (PSD) curves of samples are displayed in Figure 5. The prepared samples (S1, S1a, S2, and S2b) clearly display a type IV isotherm with the obvious H3 hysteresis loop representing mesoporous structure (Figure 5a) [38]. At a relative pressure P/P_o_ of 0 to 0.3, the amount of adsorbed nitrogen increases gradually, and the two lines of adsorption and desorption almost practically merge into one. The adsorption isotherm of the samples slowly increases with the increase of P/P_o_, whereas the BET surface areas of S1, S1a, S2, and S2b are 59, 66, 19, and 78 m^2^/g, respectively, showing an increasing trend. The PSD curves (Figure 5b) show the adsorption pore size distributions in the range of 3–100 nm with a broad peak in the range of 3–20 nm, which demonstrates the presence of a mesoporous surface according to Groen’s report [39]. Table 1 summarizes the results of the pore structure parameters.

According to the findings (Table 1), S2b has the highest BET surface area (78 m^2^/g) and a mesoporous structure, which can be attributed to the removal of water from the interlayers during the process of calcination. More active sites for adsorption may be provided by the porous hierarchical structure and high surface area.

### 3.4. FTIR Spectra

**Figure 6:**
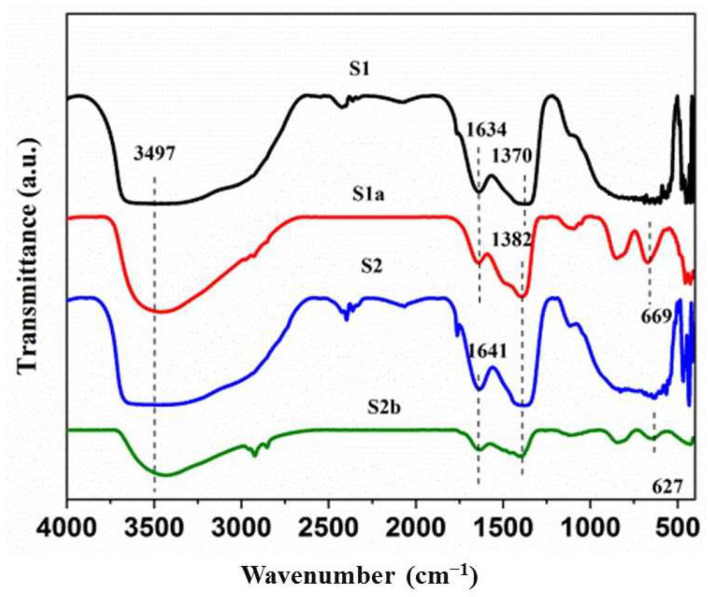
FTIR spectra of prepared samples.

Figure 6 displays the FTIR spectra of the prepared materials. The hydroxyl group present in the interlayer and the presence of water molecules on the surface can be attributed to the stretching vibrations that cause the absorption bands at 3497, 1634, and 1641 cm^−1^, respectively [20,40]. The peaks at 1370 and 1382 cm^−1^ indicate the co-existence of nitrates [35] and carbonate species [41], respectively, in the galleries of LDHs. The new broad and intense bands appear at low frequency, i.e., 627 and 669 cm^−1^ in S1a and S2b, as compared to S1 and S2, and can be ascribed to the lattice vibrational modes of M–O and O–M–O (M = Mg^2+^, Ni^2+^, or Al^3+^), as these are composed of positively charged layers of metal oxides with interlayer oxygen atoms due to the large number of metal-oxygen bonds [42], which is also in agreement with the XRD characterization. 

### 3.5. Adsorption of Methyl Orange

#### 3.5.1. Effect of Initial pH on Dye Adsorption

**Figure 7:**
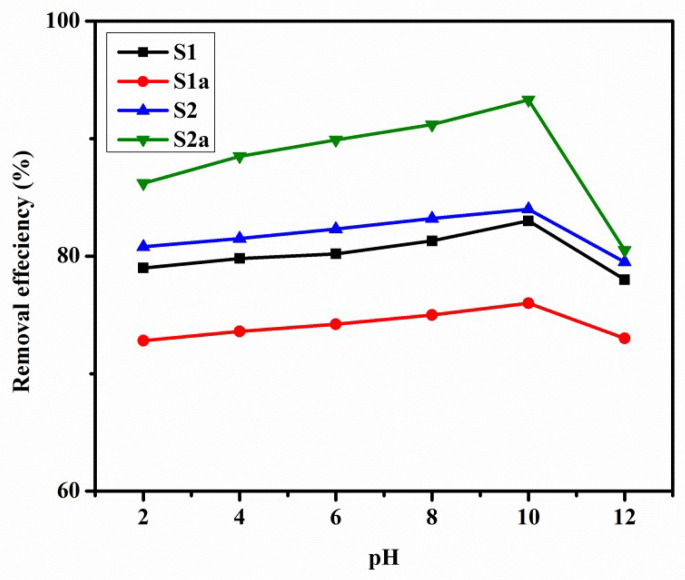
Effect of pH when MO = 25 mg/L, m = 0.01 g, V = 0.1 L, and T = 25 ± 1 °C.

The pH is an important factor in the control of the adsorption process. The effect of pH in the range of (2–12) on MO dye adsorption is shown in Figure 7. MO adsorption by synthesized samples is not significantly affected by a change in pH from 2–10. After pH 10, a reduction in adsorption was seen, which is due to the increased competition between excess OH^−^ and MO anions for the surface sites, decreasing the adsorption of MO on the surface [43,44]. 

Furthermore, the maximum adsorption capacities of samples S1a and S2b (LDOs) were higher than samples S1 and S2 (LDHs) for MO dye. This was due to the reconstruction of S1 and S2 with the interaction of MO dye with S1a and S2b in solution [45].

#### 3.5.2. Effect of Dosage

**Figure 8:**
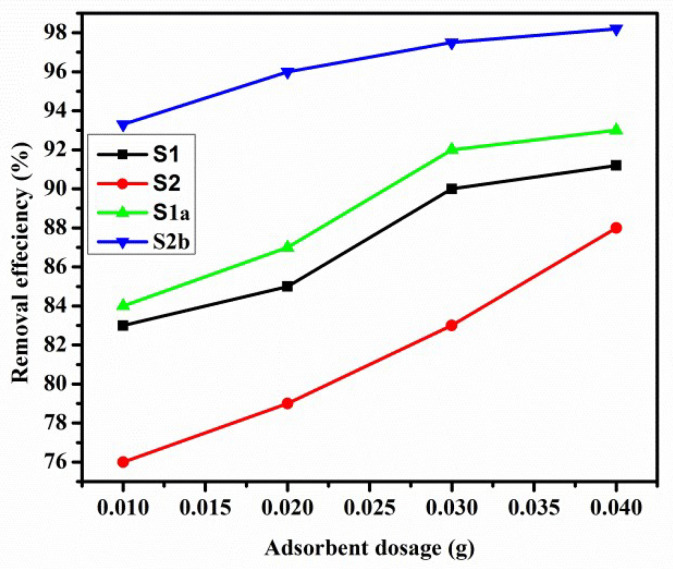
Effect of adsorbent dosage when MO = 25 mg/L, m = 0.01–0.04 g, V = 0.1 L, T = 25 ± 1 °C, and pH = 9 ± 0.2.

Figure 8 shows the effect of the dosage of adsorbents on the percent removal efficiency (%) of prepared samples. The results indicated that as adsorbent dosage was increased, the percent removal efficiency (%) increased from 83% to 91% for S1, 84% to 93% for S1a, 76% to 87% for S2, and 93% to 98% for S2b, respectively. However, the maximum amount adsorbed (mg/g) decreased. This decrease is due to the unviability of adsorption sites because of aggregation, which reduces the total surface area accessible for further dye adsorption. 

A 0.01 g sample of adsorbent dosage was chosen as the optimum dosage to perform experiments [46].

### 3.6. Adsorption Kinetics

**Figure 9:**
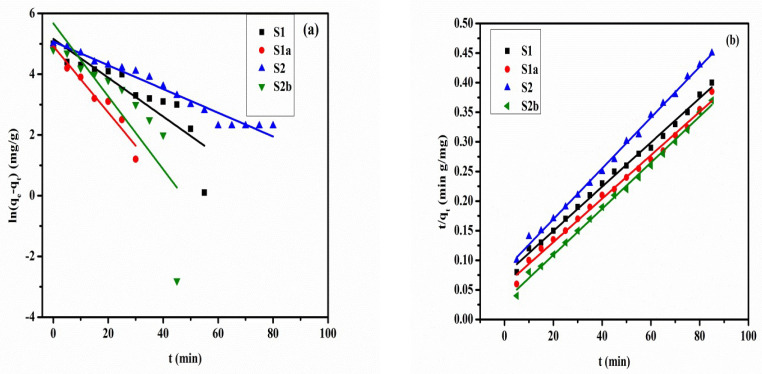

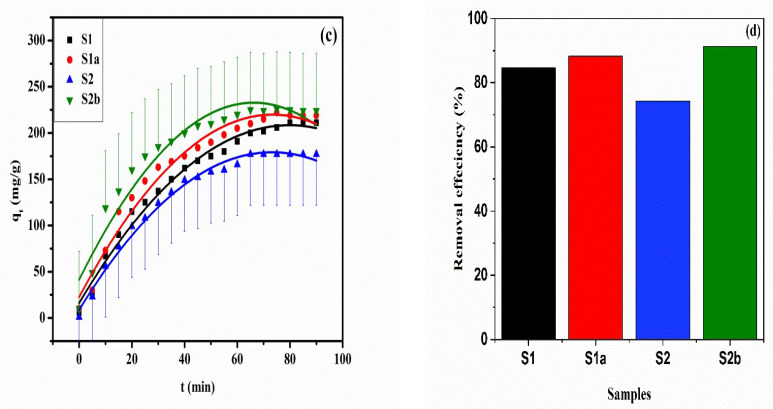
Adsorption kinetics, pseudo-first-order model (**a**), and pseudo-second-order model (**b**). Effect of time on the removal capacity of MO for prepared samples (**c**) and removal efficiency (%) of prepared samples (**d**) when MO = 25 mg/L, m = 0.01 g, V = 0.1 L, T = 25 ± 1 °C, and pH = 9 ± 0.2.

Kinetic study is a vital factor in evaluating the effect of time and the mechanism of sorption reactions [47]. The impact of contact time on MO adsorption by prepared samples S1, S1a, S2, and S2b is illustrated in Figure 9. Figure 9a,b shows the pseudo-first-order model and the pseudo-second-order kinetic model fitting the samples for the adsorption of MO dye under specific conditions. Table 2 lists the constants that were determined from the linear forms of kinetic models, which shows that The obtained correlation coefficients (R^2^) are larger than 0.9, the values of the estimated *q_e_* from the pseudo-second-order model are almost comparable to the experimental *q_e_*, and the kinetic adsorption fits better to the pseudo-second-order model. These findings clarify that the pseudo-second-order sorption mechanism predominates and that a chemisorption process appears to be in control of the overall rate constant of the sorption process. The MO removal capacity of four samples is shown in Figure 9c as a function of contact time, and the removal efficiency (%) of the samples is shown in Figure 9d. From Figure 9c, one can observe that within the first 40 min of the adsorption process, adsorption occurs continuously and speedily, and it took almost the same amount of time (80 min) for all four samples to achieve adsorption equilibrium. The low MO dye removal values found for S1 and S2 can be used to explain the strong carbonate affinity for LDH compounds. However, it is significantly higher for calcined products S1a and S2b because the adsorption is likely caused by both surface adsorption and reconstruction mechanisms [48]. The same equilibrium time for S1a and S2b for S1 and S2 is due to reconstruction phenomena (memory effect) [25].

### 3.7. Adsorption Isotherm

**Figure 10:**
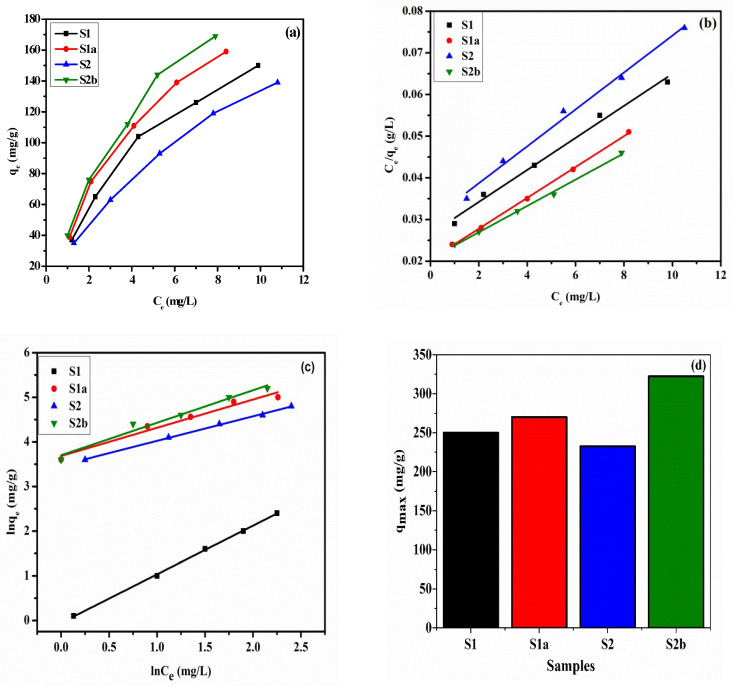
The equilibrium adsorption isotherms, effect of MO concentration on removal capacity (**a**), Langmuir isotherm model (**b**), Freundlich isotherm model (**c**), and maximum removal capacity of prepared samples (**d**) when the MO concentration was 5–25 mg/L, m = 0.01 g, V = 0.1 L, T = 25 ± 1 °C, and pH = 9 ± 0.2 (without adjusting the initial pH).

The adsorption isotherms for the uptake of MO onto S1, S1a, S2, and S2b at MO concentrations of 5–25 mg/L, m = 0.01 g, V = 0.1 L, T = 25 ± 1 °C, and pH = 9 ± 0.2 (without changing the initial pH) are shown in Figure 10a–d.

The MO dye adsorption isotherms of the four prepared samples after 80 min. of contact time are presented in Figure 10a with different MO concentrations (5 to 25 mg/L). The Langmuir isotherm theory is based on adsorbate monolayer coverage across a homogeneous adsorbent surface. The fundamental assumption is that adsorption occurs at specified, homogenous locations within the adsorbent. Once a dye molecule has occupied a site, no subsequent adsorption may occur there. Figure 10b represents the Langmuir adsorption isotherm model for all samples. The Langmuir model is appropriate for modeling the MO adsorption equilibrium onto the S1, S1a, S2, and S2b. The Langmuir isotherm-determined monolayer maximum adsorption capacity is 322.5 mg/g for S2b, as shown in Table 3. The value of R_L_ determines whether the isotherm has an unfavorable (R_L_ > 1), linear (R_L_ = 1), favorable (R_L_ = 1), or irreversible (R_L_ = 0) shape [14]. The calculated R_L_ values for various initial MO concentrations range from 5 to 25 mg/L, which lie between 0 and 1, indicating that adsorption is a useful process. Furthermore, the low R_L_ values suggested that the interaction of dye molecules with samples was quite strong [49]. The obtained values of q_max_ and K_L_ are calculated from the linear Langmuir plot. The values for Langmuir constant K_L_ (0.1410–0.1787) are within the range of 0–1, which specifies the favorable intake of MO. The isotherm correlation coefficient is relatively high (R^2^ = 0.9996), indicating that the Langmuir model is adequate for explaining the adsorption equilibrium of MO onto the prepared samples. According to Table 3, the maximal removal capacity for S1 is 250 mg/g, S1a is 270.2 mg/g, S2 is 232.55 mg/g, and S2b is 322.58 mg/g. 

After calcination, specific surface areas of S1a and S2b are increased, which in turn is responsible for producing more active sites and enhanced adsorption capacity for the pollutant. The adsorption capacity values for S1 and S2 are lower than the calcined samples. The possible reason is that it is difficult to dislocate the carbonate anion position from the interlayers of hydrotalcite compounds, as these structures have strong carbonate anion binding affinity [36]. After adsorption of MO, the adsorption isotherms of samples S1 and S2 were more dispersed due to the complexity created by intercalated ions, while the reconstructed structures of samples S1a and S2b did not include interlayer carbonate anions [50]. Furthermore, from the Langmuir isotherm model equation, the calculated q_max_ of S2b for MO is greater than that of used materials as adsorbents, as recently stated in the past work in Table 4. The inconsistency might be attributed to the unique and different structures and physical or pore properties.

The Freundlich isotherm is an empirical equation that assumes adsorption occurs on heterogeneous surfaces and that adsorption capacity is related to dye concentration at equilibrium. The plot between lnq_e_ and lnC_e_ (Figure 10c) provides the Freundlich isotherm constants K_F_ and n. The Freundlich constant n indicates the adsorption process’s favorability. For optimal adsorption conditions, n should be less than 10 and greater than unity. In this case, the value of n for the Freundlich model was greater than one, indicating that the adsorption of MO onto S1, S1a, S2, and S2b was favorable. Moreover, the correlation coefficient (R^2^ = 0.991) indicates that the experimental data coincide with the Freundlich model. The isotherm parameters investigated for the Langmuir and Freundlich models are presented in Table 3. 

The evaluated values of R_L_ are in the range of 0–1 (Table 3), which illustrates the efficient adsorption of MO by NMA samples [43].

### 3.8. Regeneration Cycles

**Figure 11:**
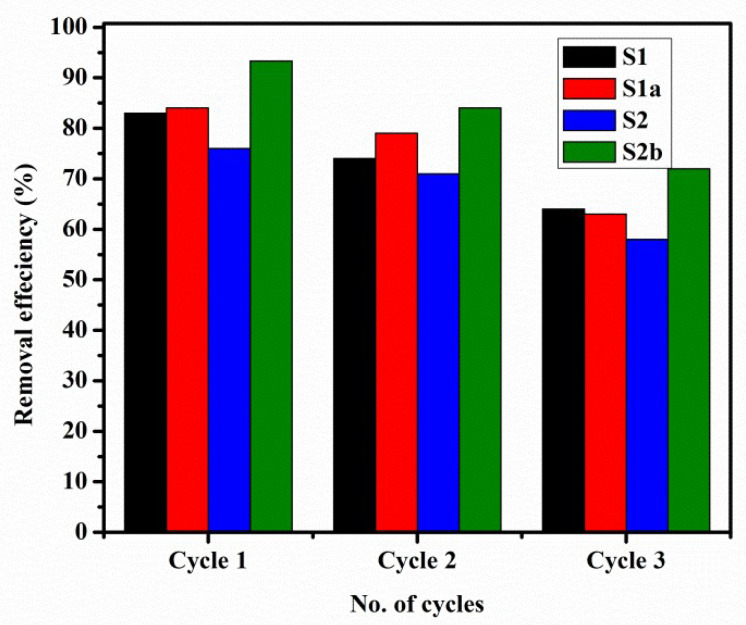
Reusability of adsorbents for the adsorption of MO in three adsorption-desorption cycles when MO = 25 mg/L, m = 1 g, V = 0.1 L, T = 25 ± 1 °C, and pH = 9 ± 0.2.

Regeneration tests were carried out in order to assess the effectiveness, stability, and possibility of repeated application of the prepared samples. The removal of MO from the adsorbent after each cycle was performed by using 8% HNO_3_ as a desorption agent. Figure 11 displays the removal efficiency (%) decrease from cycle 1 to cycle 3. In the first cycle, the percentage adsorption for adsorbents S1, S1a, S2, and S2b was 83%, 84%, 76%, and 93.3%, respectively. Whereas 62%, 63%, 58%, and 72% removal percent were observed for adsorbents S1, S1a, S2, and S2b, respectively, in the third cycle. This decrease in removal percent can be attributed to the influence of surface area and the gradual collapse of the porous structure into a more compact structure [30]. The feasibility of reuse for the prepared samples with complete adsorption of MO dye molecules has been established, which is useful in its practical waste water treatment applications [44].

## 4. Conclusions

We synthesized Mg/Al LDH by the urea method and converted it into Mg/Al LDOs by roasting it at 400 °C. These metal oxides reformed (memory effect) finally into tri-metallic Ni/Mg/Al layered double hydroxides (NMA-LDHs) when we added Ni in different percentages (S1 and S2) by the co-precipitation method. S1 and S2 were changed into Ni/Mg/Al layered double oxides (S1a and S2b) after being calcined at 400 °C. These prepared samples have a hierarchically porous structure, with the lamellar sheet’s morphology having various pore densities. The characteristic XRD studies accessible in this work are accompanied by valuable mechanistic and kinetic interpretations. The adsorption performance of MO is strictly related to the feeding ratio of Ni^2+^ in NMA-LDOs, as the insertion of nickel ions into the LDH lattice results in additional surface defects and positively charged sites, which increase MO’s chemical affinity. The S1, S2, S1a, and S2b have mesoporous structures, which account for the greater specific surface area and broader pore size dispersion (3–100 nm). The calcined samples (S1a and S2b) showed a large percentage of MO removal (84% and 93%, respectively) as compared to the uncalcined samples (S1 and S2). The optimal dosage and contact time of prepared samples were 0.01 g and 80 min, respectively. pH was found to have an insignificant effect on the adsorption capability of all samples in the pH range of 2 to 10. The adsorption kinetics of all samples adsorbing MO were established to follow the pseudo-second-order model. Both the Langmuir and Freundlich models were well-fitted to the data, with Langmuir adsorption capacity following the order: S2b > S1a > S1 > S2. Furthermore, the adsorption-desorption cycles indicate better reusability and stability of the prepared samples. All outcomes showed that NMA-LDHs and NMA-LDOs were efficient and cheap alternatives for the removal of dyes from wastewater.

## Figures and Tables

**Table 1:** Pore diameter, pore volume, and BET surface area of samples.

Samples	d_pore_ (nm)	V_pore_ (cm^3^/g)	S_BET_ (m^2^/g)
S1	15.26	0.001	59
S1a	19.92	0.001	66
S2	15.77	0.002	19
S2b	23.90	0.003	78

**Table 2:** Pseudo-first-order and pseudo-second-order kinetic model constants of the as-prepared samples.

Samples	q_e,exp_ (mg/g)	Pseudo-First-Order Model			Pseudo-Second-Order Model		
		q_e,cal_ (mg/g)	k_1_ (×10^−2^ min^−1^)	R^2^	q_e,cal_ (mg/g)	k_2_ (×10^−3^ g/mg min)	R^2^
S1	211.4	1.636	7.47	0.9043	246.0	2.51	0.970
S1a	220.6	1.646	7.54	0.7531	250.0	4.50	0.991
S2	185.5	1.483	3.70	0.9700	222.2	2.82	0.972
S2b	228.3	1.719	6.70	0.8166	256.4	2.89	0.980

**Table 3:** Langmuir isotherm and Freundlich isotherm model constants of the as-prepared samples.

Samples	Langmuir Isotherm Model				Freundlich Isotherm Model			
	q_max_ (mg/g)	K_L_ (L/mg)	R^2^	R_L_	q_max_	K_F_ (mg/g) (L/mg)^1/n^	n	R^2^
S1	250.0	0.1562	0.993	0.169–0.359	5.674	1.285	1.55	0.991
S1a	270.2	0.1787	0.999	0.156–0.528	5.130	1.320	1.54	0.988
S2	232.5	0.1410	0.993	0.154–0.369	5.620	1.248	1.57	0.991
S2b	322.5	0.1527	0.993	0.171–0.567	5.813	1.331	1.41	0.985

**Table 4:** Comparison of the removal capacities of different adsorbents for MO removal.

Adsorbents	Surface Area (m^2^/g)	q_max_ (mg/g)/Removal%	References
Zn/Al-LDO	-----	181.9	[1]
C-NiAl	90	291.9	[38]
NiFe-LDH	-----	246.9	[43]
CaAl-LDH	-----	96.6	[51]
Mg/Fe-LDH	48.7	194.9	[27]
Ni/Mg/Al-LDO	77.8	322.5	This work

## Data Availability

All the data is enclosed in the manuscript.
